# Round the Hospitals

**Published:** 1918-10-05

**Authors:** 


### ROUND THE HOSPITALS.

In The Hospital of March 23, page 534, we called attention for the second time to the fact that nurses frequently have relatives dependent upon them, and inquired whether nurses on military service would be entitled to any Government pension available to relieve the distress caused by the death of the breadwinner. Sir Archibald Williamson, Bart., M.P., a member of the Council of the Royal National Pension Fund for Nurses, raised the same point in the House of Commons, and asked the Government whether Army nurses were eligible for pensions if they suffered injury through the war, and, if so, would steps be taken to place them on the same footing as other branches of the Army by making provision for dependents in cases where a nurse lost her life. No cases of relatives dependent on Army nurses had at that time been brought to the notice of Mr. Hodge, the Minister of Pensions. We have the pleasure to announce, on the authority of a letter kindly sent us by Sir Archibald Williamson, and through the Ministry of Pensions, that approval has recently been given to treating for purposes of pension the relatives of members of the Nursing Services on lines similar to those followed in the case of relatives of officers, subject to certain conditions such as dependence and pecuniary circumstances. The Treasury sanction has been obtained to the granting of these pensions to nurses' dependent relatives, and the whole nursing profession and all who are interested in our nurses—and who is not?—will not only welcome this just concession, but will acknowledge their sense of indebtedness to Sir Archibald Williamson, who is an able and busy member, full of work of national importance, which makes his action the more noteworthy and grateful, for his prompt intervention and success in obtaining the grant of these pensions. Trained nurses owe a deep debt of gratitude to every member of the Council of the Royal National Pension Fund for Nurses, who include in their number some of the most able, representative and important financiers known to the City of London, who have from the first given yeoman service and much time and thought to the work of the Pension Fund as an act of personal service and thanksgiving for the blessings of health. May we ask every nurse reader: Have you yet sent your shilling contribution to the Junius S. Morgan Benevolent Fund in connection with the Royal National Pension Fund? If not, please make a point of seeing that one shilling from yourself reaches Mr. Dick for that Fund at 15 Buckingham Street, Strand, W.C., before the week is out.

The excellent gathering of nurses who assembled at 3 Vere Street on September 25, to discuss the advisability of founding a London Centre of the College of Nursing attacked the business before them with zeal and determination. Miss A. C. Gibson was in the chair and had the pleasant duty of presiding over an entirely unanimous meeting. Sir Arthur Stanley was able to tell the nurses that a vast deal of spade work had already been accomplished by the College, besides the enrolment of over 10,000 nurses. "Innumerable obstacles and questions which would have raised debate in the House of Commons" have been got rid of, and the goal of State Registration is appreciably nearer at hand. The College is shortly moving out of the premises at 6 Vere Street hitherto placed at its disposal rent free by Sir James Boyton and supplemented by arrangements with the College of Ambulance. The time is therefore ripe for the formation of a London Centre within which some of those most intimately concerned with building up the future of the College may learn to develop their ideas.

---

The value to the College of the decentralisation system now at work in the formation of Local Centres was vigorously brought home to the meeting by Miss Sparshott, R.R.C. She spoke inspiring words of the work already set on foot in the Manchester Centre, and urged on Londoners the importance of establishing the Centre on a sound financial basis. That Manchester should set the example which London is now enthusiastically following is a fine illustration of the unity which already begins to make itself felt in the nursing profession. Miss Coode wisely laid stress on the need for each individual nurse's influence and effort in moulding the profession at large. Keenness and zest in everyone concerned are essential to make any Centre a complete success. There is no reason to think that London nurses will be found wanting.

---

The scheme for the formation of a London Centre was introduced to a general meeting, elected for that purpose out of the members present, by Miss Rundle. The Executive Committee of the London Centre comprises some very well known names, including Miss A. C. Gibson and Miss Ray, both of whom, it is hoped, may find time to devote some of their long-accumulated wisdom and practical knowledge of nursing affairs to the development of the London Centre of the College. For *Local Representative* the Executive Committee have been fortunate in securing Miss Mont-

**ROUND THE HOSPITALS**—(*continued*).

gomery, matron of the Middlesex Hospital. The *Honorary Secretary* is Miss Biggar, sister, St. Thomas's Hospital, and the *Honorary Treasurer* Miss Sordy, matron of Queen Mary's Hospital for the East End. Under such promising auspices the London Centre begins its career, the Executive Committee being charged with the duty of making arrangements to secure the all-important club-rooms round which the activities of the Centre may have full play.

The next step to the formation of a vigorous Centre is for all members of the College of Nursing who reside in or near London to send in their names as members to the Honorary Secretary, Miss Biggar, St. Thomas's Hospital. The annual subscription has been fixed at 5s., which, as including use of club-rooms, must be reckoned moderation itself. Members will greatly facilitate the task of the Honorary Secretary by enclosing the subscription for the first year with their application for membership, and by writing distinctly and giving, if possible, their registration number. It ought not to be difficult, even in times like these, to enrol a thousand names within a short time.

The new Cambridge Centre of the College of Nursing held its first meeting on September 25 for the purpose of appointing committees and officers. A very good representative gathering assembled at Addenbrooke's Hospital, and the following honorary officers were elected: *Chairman and Local Representative*, Miss C. C. Crookenden, matron of Addenbrooke's Hospital; *Treasurer*, Miss W. Flint, sister, King's College, Cambridge; *Secretaries*, Miss E. Armitage, matron, County Hospital, Huntingdon, and Miss K. Newman, sister, Addenbrooke's Hospital. The General and Executive Committees were also appointed. The subscription for members of the Centre has been fixed at 2s. 6d., and the financial year starts on October 1. All members of the College in Cambridge and the surrounding districts are requested to communicate with one of the honorary secretaries, stating to what branch of nursing they belong. The Executive Committee is holding its first meeting as we go to press on October 2, at four o'clock, in Addenbrooke's Hospital, and the programme for the winter session will then be arranged.

The devotion of the Canadian nurses in recent air raids has been recognised by the award of the Military Medal to the following matron and sisters for distinguished service in the field: Matron Edith Campbell, R.R.C., who, regardless of personal danger, attended to the wounded sisters and by her personal example inspired the sisters under her charge; Sister Leonora Herrington, who remained on duty the whole night and by her excellent example and personal courage was largely responsible for the maintenance of order and discipline; Sister Lottie Urquhart, who, when four bombs fell on her wards, attended to the wounded regardless of danger and gave an inspiring example of courage and devotion to all; Sister Janet Mary Williamson, who displayed exceptional coolness in a badly damaged ward, sustained her patients, and ensured their evacuation; Sister Meta Hodge and Sister Eleanor Jean Thompson, who, although both injured by a falling beam, displayed great presence of mind in extinguishing overturned oil-stoves and rendered valuable assistance in the removal of patients. All these belong to the Canadian Army Medical Corps or the Nursing Service, on which they have shed fresh lustre.

At the Investiture held at Buckingham Palace on September 25 the King conferred the decoration of the Royal Red Cross (Second Class) on Sister Jane Trotter, Q.A.I.M.N.S.(R.); Sister Lavinia Taylor, T.F.N.S.; Miss Mary Fynes-Clinton, the Lady Arthur Grosvenor, and Miss Margaret Richardson, V.A.D.

We are asked to state that nurses holding certificates for three years' general training who are desirous of joining Queen Alexandra's Imperial Military Nursing Service Reserve for duty in military hospitals should apply in writing, without delay, to the Matron-in-Chief, Queen Alexandra's Imperial Military Nursing Service, War Office, Adastral House, Victoria Embankment, E.C.

The denial of the title of "nursing sister" to staff-nurses of the Territorial Force Nursing Service brings forcibly to notice the inadequacy of the term "nurse" to describe a fully qualified member of the profession. It is regrettable that the T.F.N.S. does not accord the term "nursing sister" to trained women who under the title of "staff-nurse" are doing sisters' work. But this is only part of an anomaly from which the whole nursing world, not merely a section of it, is suffering. There is at present no generally recognised term for a fully trained and certified member of the nursing profession. We have before now urged that it should be part of the movement in favour of State Registration to secure for the registered nurse, and for her only, the title of "nursing sister." We fear there is very little to be gained at this juncture of affairs by kicking against the pricks of Army regulations. Better to reserve all energy for the struggle to gain State recognition.

A disgraceful affray has recently occurred at the Belmont Road Military Auxiliary Hospital, Liverpool, in which an Irish nurse, Miss McShane, was killed. The hospital contains both coloured and white men—a bad arrangement, to begin with—and the former have been getting out of hand in their convalescent stage. The military police guard endeavoured to tighten up the relaxed discipline, with the result that several of the West Indian men ran amok, and a free fight ensued among the patients, the wounded British soldiers of white race coming to the rescue of the authorities. Nurse McShane, who pluckily intervened to minister to one of the laundrymaids, was caught in the rush and knocked down. Though apparently

**ROUND THE HOSPITALS**—(*continued*).

not seriously injured, she developed pneumonia, and died within a few days. Had the coloured patients been under the control of officers accustomed to their racial characteristics the incident could never have happened. English disciplinary methods applied to these men are foredoomed to excite discontent and revolt, and the authorities must be held gravely to blame for allowing matters to come to this pitch.

The result of the Mitcham Hospital strike must appear to the impartial observer as deplorable. The Board was seriously at fault in deferring consideration of admitted grievances till after the holidays, and it cannot be too clearly understood that ninety-nine out of every hundred labour disputes arise from the shirking and dilatory attitude adopted by employers towards appeals for better conditions among their workers. But to afford such a signal exhibition of the success of the strike weapon in a hospital for wounded soldiers was pitiable weakness. It should certainly not have proved impracticable, under a higher rate of pay and with shorter hours, to replace the entire staff found capable of threatening the wounded, for that is what it amounted to.

The Nurses' Missionary League holds its autumn reunion on October 2, and is making plans for a great recruiting campaign early in the spring. From all parts of the world the call comes for earnest-minded workers, and no class of workers is needed more than nurses qualified to lead and instruct the women of other races whose eyes are turning towards the light. Among the vast numbers of nurses set free for other work when the war closes there will doubtless be found many admirable recruiters and recruits. Miss Jolley, Matron-in-Chief, R.A.F. Nursing Service, is one of the hostesses at the afternoon conversazione, with Miss Bennett, matron of the Metropolitan Hospital, and Mrs. Drummond Robinson (London Hospital).

Most alarming accounts are coming through of the cholera epidemic in Petrograd, which is adding to the horrors of the revolution. There appears to be no organised attempt to combat the disease. The filth and disorder of the lazarettos and hospital wards are altogether beyond belief. The few doctors who are available are thwarted in every way as "suspect" by the Bolshevik authorities, and are under the dominion of the patients and attendants. Many sisters and nurses have died of the disease, and of systematic nursing there appears to be no trace. Drugs are for the most part unobtainable. Groups of the bourgeois class, men and women, are being commandeered to dig graves and bury the dead. The only crumb of comfort is that the virulence of the epidemic is wearing itself out, and the victims have sunk from 900 a day to fifty or sixty. But those whom cholera spares are exposed to the equally deadly risk of death from starvation.

A little pamphlet [F.S.B. 22] likely to be of great service to institution housekeepers has been issued by the Dispatch Department of the Ministry of Food, 35 Park Street, London, W. 1. It gives a collection of recipes suitable for institution use in the present days of shortage and the correct formula for large numbers. Every recipe gives the quantity of each ingredient needed for so many helpings, and the value to the cook of these calculations is apparent. Some of the dishes are distinctly original, and all are practical and inexpensive. Housekeepers should apply at once for a copy.

In spite of the increased price of milk and the fear of a scarcity in some districts, the *National Food Journal*, official organ of the Ministry of Food, is urging its immense importance from the dietary point of view and the desirability of an all-round increased consumption by the public. Its advantage to the housewife in that it is a food in which there is no waste at all, every drop contributing nourishment if carefully preserved, is well put. For hard workers, particularly such as live indoors, it is irreplaceable. In most institutions the supplies of milk have of late been strictly curtailed. But we believe this form of economy may be carried too far. Where a lessening of the vitality is observable on war rations, the balance may be restored by as little as an extra ¼-pint of milk daily. Independently of its protein, fats, and sugar, the vitamines which it contains render the rest of the diet more nourishing and promote the building of tissue.

An illuminating report from the State Children's Association, of which Mrs. S. A. Barnett, C.B.E., is hon. secretary, exposes the wide field of work and need for workers in this department of national life. With a total of 186,346 children supported by the rates, under varying conditions, nearly 10,000 of whom are still in the workhouses, the need for a quickened sense of public responsibility in respect of these little wards of the nation is evident. But it is in the matter of the child delinquent that the State Children's Association is most insistent on reform. The number of children and young persons brought before the Juvenile Courts has increased by over 14,000 in the last three years of war, and the stultifying practice of sending children between the ages of fourteen and sixteen to prison is still common. England has much to learn from the United States in regard to juvenile delinquency. The intimate connection between the nurse's work and the magistrate's is brought into view by the practice at Denver, U.S.A., of medically examining each child brought before the court, both as to mind and body. Often some physical defect is at the root of the delinquency. Often some medical and nursing treatment or slight operation, performed by consent of the parents, is all that is necessary to turn a young offender into a well-behaved girl or boy. Two hundred such cases in two years is a wonderful average, and opens up a new line which even the most old-fashioned martinets must approve. Our readers should write to 53 Victoria Street, S.W. 1, for a copy of this report.

